# Retinal arteriolar occlusions due to cytomegalovirus retinitis in elderly patients without HIV

**DOI:** 10.1186/1869-5760-3-17

**Published:** 2013-01-21

**Authors:** Janet L Davis, Payman Haft, Kristen Hartley

**Affiliations:** 1 Bascom Palmer Eye Institute, University of Miami Miller School of Medicine, 900 NW 17th ST, Miami, FL 33136, USA

**Keywords:** Cytomegalovirus retinitis, Immunocompetent, Immunocompromise, Aged, HIV, Retinal arteriolar occlusion

## Abstract

**Background:**

Five of 7 (71%) elderly immunocompetent patients with cytomegalovirus retinitis had retinal arteriolar occlusions versus 2 of 8 (25%) elderly immunocompromised patients and 1 of 19 (5%) younger HIV-infected patients. Compared to HIV-infected patients, elderly patients were more likely to have occlusive events, neovascularization or hemorrhage, and underlying vasculopathy. The purpose of this study is to report the novel finding of extensive retinal arteriolar occlusions and neovascularization in immunocompetent patients with cytomegalovirus retinitis. This is a retrospective observational cohort study of cytomegalovirus retinitis (CMVR) in a university setting. Seven patients were elderly but not immunocompromised, 8 were elderly and iatrogenically immunocompromised, and 16 were HIV-infected. All patients underwent polymerase chain reaction testing of intraocular fluid. Primary outcome measure was visual acuity. Secondary outcome measures were vascular occlusions, ischemic complications, and response to treatment.

**Results:**

Mean age was 73, 70, and 41 years for immunocompetent, immunocompromised, and HIV-infected patients, respectively. Diabetes and vascular disease were common in the elderly. Vision loss to less than 5/200 occurred in 50% of the immunocompetent elderly patients, and 17% of CMV eyes in immunocompromised and HIV patients. Occlusion of the entire retinal vasculature occurred in 4/7 (57%) of immunocompetent patients despite lack of Zone I involvement, and rubeosis occurred in three, disc neovascularization in one, and vitreous hemorrhage in two patients. Vascular occlusive events were less common in immunocompromised patients and rare in the HIV-infected.

**Conclusions:**

CMVR in non-HIV-infected elderly patients is associated with retinal arteriolar occlusions. An intact host immune response may increase damage to retinal vessels. Prompt diagnosis may avert catastrophic vision loss.

## Background

Cytomegaloviral infection is common. Seroprevalence increases steadily in the USA from 36.3% in 6 to 11-year-olds to 90.8% of those aged 90 or older [1]. The most common ocular manifestation of cytomegalovirus infection is cytomegalovirus retinitis (CMVR), which typically occurs as a resurgent infection in a latently infected host who is severely immunocompromised by HIV infection, chemotherapy, or organ transplantation. CMVR can also rarely present in the elderly or in other minimally compromised individuals as an atypical acute retinal necrosis (ARN) [2-4]. Recognition of cytomegalovirus infection is important as specific anti-CMV treatments rather than the anti-viral agents used for other herpes class viruses are required to control the infection.

We present cases of CMVR in 15 elderly, non-HIV-infected adults in order to facilitate clinical recognition and to expand the current literature. Eight of the 19 affected eyes had devastating visual outcomes after delays in diagnosis; in 5 of the 8 eyes, this was due to retinal arteriolar occlusion, a previously under-recognized complication of CMVR. The frequency of retinal vascular occlusion was much higher than in younger HIV-infected patients with CMVR.

## Results

Fifteen patients age 60 and older were identified who were not HIV-infected and had a diagnosis of CMVR after diagnostic PCR testing. Seven, with an average age of 73 ± 7.4 years, were judged to be immunocompetent. Of these, one was taking low doses of oral corticosteroids for healed giant cell arteritis, and four had controlled diabetes mellitus; one had chronic lymphocytic leukemia (CLL) that was controlled with no medication (Table 1). Eight patients, with an average of 72.2 ± 12.2 years, were judged to be immunocompromised. Of these, two were organ transplant recipients, and two had lymphoma being treated with chemotherapy; the remaining patients were receiving immunosuppression for a variety of autoimmune disorders (Table 2). Sixteen HIV-infected patients with CMVR were selected for comparison because the diagnosis had been confirmed with PCR, and they were contemporaneous (2000–2005) with the non-HIV-infected patients. Average age was 40.3 ± 11.6 years.

**Table 1:** Clinical characteristics of seven elderly, immunocompetent, non-HIV-infected patients with cytomegalovirus retinitis

**Case**	**Age/sex/eye**	**Concurrent illnesses/therapy**	**Months from onset to RAO**	**Retinitis extent at RAO zone (clock hours)**	**Diagnostic method**	**Vascular event (vision in affected eye)**
1	71/M/OD	DM, HTN subtenon injection of triamcinolone	5	Zone II-III (2)	Aqueous PCR+ CMV, retinal biopsy with cytomegalic inclusion bodies	CRAO (LP)
2	81/M/OD	MS, CVA, DM, hyperlipidemia	2	Zone II-III (3)	Aqueous PCR+ CMV	CRAO (HM)
3	66/M/OS	POAG, CLL on no treatment	2	Zone II (1)	Vitreous PCR negative, aqueous PCR+ CMV	CRAO (HM)
4	77/F/OD	PMR, GCA, prednisone 10 mg, subtenon triamcinolone	4	Zone III (1)	Vitreous PCR negative, response to ganciclovir	CRAO (LP)
5	61/M/OD	CHF, HTN, DM, aortic aneurysm	4	Zone II (5)	Aqueous PCR+ CMV	BRAO (20/30)
6	80/F/OD	HTN	NA	Zone III (1)	Aqueous PCR+ CMV	Zone I arterial sheathing (20/30)
7	75/M/OD	DM, CAD	NA	Zone I-III (9)	Aqueous PCR+ CMV	None (HM)

BRAO, branch retinal arteriolar occlusion; CAD, coronary artery disease; CHF, congestive heart failure; CLL, chronic lymphocytic leukemia; CMV, cytomegalovirus; CRAO, central retinal artery occlusion; CVA, cerebral thromboembolic event; DM, diabetes mellitus; F, female; GCA, giant cell arteritis; HM, hand motions; HTN, hypertension; LP, light perception; M, male; MS, multiple sclerosis; NA; not applicable; OD, right eye; OS, left eye; OU, both eyes; PCR+, positive result by polymerase chain reaction; PMR, polymyalgia rheumatica; POAG, primary open angle glaucoma; RAO, retinal arteriolar occlusion; Zone, anatomic area of retina involved with retinitis; Zone I, arcade to arcade with 1-disc diameter border around optic nerve; Zone II, arcades to equator; Zone III, equator to ora.

**Table 2:** Clinical characteristics of eight elderly, immunocompromised, non-HIV-infected patients with cytomegalovirus retinitis

**Case**	**Age/sex/eye**	**Concurrent illnesses/therapy**	**Months from onset to RAO**	**Retinitis extent at RAO zone (clock hours)**	**Diagnostic method**	**Vascular event (vision in affected eye)**
8	83/M/OD	DM, renal transplant, azathioprine, methylprednisolone, shingles	0	Zone I-III (3)	Aqueous PCR+ CMV	CRAO (1/200)
9	60M/OD	Myasthenia gravis, corticosteroids, cyclophosphamide, DM, CD4 + T lymph 72, peripheral vascular disease	NA	Zone II-III (3)	Aqueous PCR+ CMV	None (20/40)
10	60/F/OD	DM, pemphigus vulgaris, corticosteroids, IVK	NA	Zone I-III (6)	Aqueous PCR + CMV	HRVO 11 months before CMVR (HM)
11	63/F/OU	Systemic vasculitis, cyclophosphamide, corticosteroids	NA	OD Zone I-III (3), OS Zone I (1)	PCR + CMV	None (OD 20/50, OS 20/20)
12	89/M/OS	Hypersensitivity pneumonia, corticosteroids, steroid-induced DM, CAD	NA	Zone III (4)	Aqueous PCR+ CMV	Zone I sheathing without occlusion (20/30)
13	70/F/OU	CLL, chemotherapy, BMT	NA	OD Zone II (1), OS Zone I-III (2)	Aqueous PCR+ CMV	None (OD 20/30, OS CF)
14	87/M/OU	Kidney transplant, cyclosporine, prednisone	NA	OD Zones II-III (1), OS Zone II-III (1)	Aqueous PCR+ CMV	None (20/40 OU)
15	66/M/OU	Lymphoma, chemotherapy	NA	Zone I-III (OD 6, OS 4)	Aqueous PCR+ CMV	None (OD 20/20, OS 20/60)

BMT, bone marrow transplant; CAD; coronary artery disease; CF, counting fingers; CLL, chronic lymphocytic leukemia; CMV, cytomegalovirus; DM, diabetes mellitus; F, female; HM, hand motions; IVK, intravitreal triamcinolone acetonide; M, male; NA, not applicable; OD, right eye; OS, left eye; OU both eyes; PCR+, positive result by polymerase chain reaction; RAO, retinal arteriolar occlusion; Zone, anatomic area of retina involved with retinitis; Zone I, arcade to arcade with 1 disc diameter border around optic nerve; Zone II, arcades to equator; Zone III, equator to ora.

Males accounted for approximately 70% of the patients in each group (*P* = 0.56). None of the immunocompetent patients had bilateral disease (0%), but 4 of the 8 (50%) immunocompromised non-HIV-patients had bilateral disease, as well as 4 of the 16 (25%) HIV-infected patients (*P* = 0.6). Profound, permanent vision loss worse than 20/400 occurred in 8 of the 19 eyes in the elderly, and 10 of the 20 eyes in the HIV-infected (*P* = 0.14). In the eight cases of profound vision loss in the elderly, five were due to arteriolar occlusion, two to CMVR; one other case had both prior vein occlusion and extensive retinitis. Among HIV-infected patients, vision loss was attributable to arteriolar occlusion in only one case (*P* < 0.001). The remaining vision loss was due to retinal detachment or to CMVR.

More arteriolar occlusions occurred in the eyes of the elderly, immunocompetent patients than in those of elderly, immunocompromised patients (5 of 7 (71%) vs. 1 of 12 eyes (8%)), *P* = 0.07. When arteriolar occlusions in non-HIV-infected patients were compared to HIV-infected, the results were highly statistically significant (6 of 19 eyes (32%) vs. 1 of 20 eyes (5%)), *P* < .001. Findings associated with ocular ischemia, such as neovascularization, rubeosis, and vitreous hemorrhage, were also more common in non-HIV-infected patients (6 of 19 eyes (32%) vs. 4 of 20 eyes (20%)), *P* < 0.003.

Vasculopathic diagnoses such as cardiovascular disease and diabetes were more common in the elderly, especially those who were not considered to be otherwise immunocompromised (Tables 1 and 2). Compared to HIV-infected patients, vasculopathy was present in 10 of the 15 patients (67%) vs. 1 of 20 (5%), *P* < 0.001. Retinal detachment was more common in eyes in HIV-infected patients than in non-HIV-infected patients (7 vs. 2, *P* = 0.03). Table 3 lists the medical and surgical interventions, and visual and anatomic outcomes.

**Table 3:** Medical and surgical interventions in elderly, immunocompetent, and elderly immunocompromised non-HIV-infected patients with cytomegalovirus retinitis

**Case**	**Medical treatment**	**Surgical interventions**	**Retinovascular disease**
1	Intravenous ganciclovir	Diagnostic paracentesis, diagnostic vitrectomy, retinal biopsy	Disc neovascularization, rubeosis iridis, ectropion uvea, vitreous hemorrhage
2	Oral valganciclovir, intravitreal foscarnet, intracameral bevacizumab	Diagnostic paracentesis, PRP	Rubeosis iridis
3	Clarithromycin, atovoquone, valacyclovir, intravenous and oral ganciclovir	Diagnostic vitrectomy, trabeculectomy, diagnostic paracentesis, glaucoma drainage device	Rubeosis iridis, neovascular glaucoma
4	Prednisone, subtenon triamcinolone, valacyclovir, ganciclovir drug delivery device	Diagnostic vitrectomy, CE/IOL	Vitreous hemorrhage
5	Intravitreal ganciclovir and foscarnet, valganciclovir	Diagnostic paracentesis	None
6	Intravitreal ganciclovir and foscarnet, intravenous ganciclovir	Diagnostic paracentesis	Sclerotic arterioles in Zone I
7	Intravitreal ganciclovir and foscarnet, valganciclovir	Diagnostic paracentesis, RD repair	None
8	Lost to follow-up	Prior diagnostic vitrectomy and PRP, diagnostic paracentesis	Vitreous hemorrhage
9	Intravitreal ganciclovir and foscarnet, valganciclovir	Diagnostic paracentesis	None
10	Valganciclovir	Diagnostic paracentesis	None
11	Valganciclovir, intravitreal ganciclovir and foscarnet	Diagnostic paracentesis	None
12	Valganciclovir, intravitreal ganciclovir and foscarnet	Diagnostic paracentesis	Vitreous hemorrhage
13	Intravenous ganciclovir	Diagnostic paracentesis	None
14	Intravenous ganciclovir, intravitreal ganciclovir and foscarnet	Diagnostic paracentesis RD repair	None
15	Intravenous ganciclovir, intravitreal ganciclovir and foscarnet	Diagnostic paracentesis	None

CE/IOL, cataract extraction with intraocular lens implantation; RD, retinal detachment; PRP, panretinal photocoagulation.

## Case reports

### Case 1

A 71-year-old Indian man with hypertension and diabetes had blurred vision in the right eye for 3 months. Vision was 20/40+ in the right eye; iritis and vitreous inflammation were present. Peripapillary and macular cotton-wool spots were noted and there was peripheral nonperfusion with scattered intraretinal hemorrhages temporally in the right eye. Diabetic retinopathy was not present. The left eye was normal.

Fluorescein angiography at that time showed intact choroidal filling and delayed filling of the arteriolar vasculature in the right eye with incomplete filling in the midperiphery. Subtenon triamcinolone injection was given elsewhere to treat the inflammation. Toxoplasmosis IgM and IgG antibodies, hepatitis B and C antibodies, serum immunoelectrophoresis, antidouble-stranded DNA, hemoglobin A1C, and antinuclear antibody were negative or normal. Serum herpes simplex virus (HSV) 1 and 2 IgG were highly positive.

**Figure 1:**
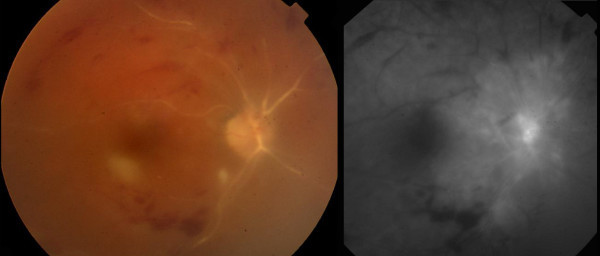
**Case 1, posterior fundus, right eye. **Left frame: there are sclerotic central vessels and cotton-wool spots located inferotemporal to the optic disc and fovea. Hemorrhages are located along the superior and inferior vascular arcades. Right frame: late-phase fluorescein angiography shows minimal retrograde filling from the optic nerve circulation of the retinal vasculature with late intraretinal staining around the optic nerve. There is extensive capillary nonperfusion peripherally.

**Figure 2:**
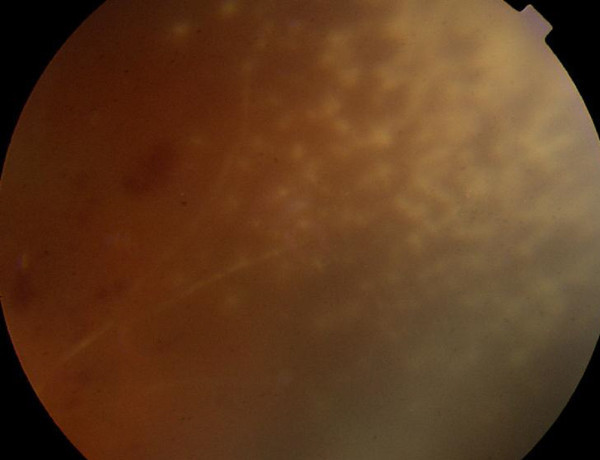
**Case 1, superonasal retina, right eye. **There are nummular patches of a granular necrotizing retinitis. A sclerotic arteriole enters the area. There are scattered intraretinal hemorrhages proximal to the optic nerve head.

Two months later on referral, visual acuity in the right eye was 5/200 with eccentric fixation. There was 3+ anterior chamber cell and 1+ flare with small keratic precipitates and 3+ vitreous cell with moderate haze. Intraocular pressure was 19 mmHg. Prominent optic nerve neovascularization was noted, and arterioles appeared sclerotic and sheathed. Dispersed intraretinal hemorrhages and a few cotton wool patches were present in the posterior pole. Repeat FA showed lack of perfusion in the area of retinal infiltration and extensive closure of the central retinal vasculature with macular nonperfusion (Figure 1). Nummular retinal infiltrates were seen in the superonasal quadrant, extending from the equator to the periphery (Figure 2).

Lab testing including complete blood count (CBC), anti-neutrophil cytoplasmic antibody (ANCA), rapid plasma reagin (RPR), fluorescent treponemal antibody, human T-cell lymphotropic virus-1 antibody, leptospira antibody, and human immunodeficiency virus (HIV) antibody were negative or normal. PCR analysis of the aqueous humor was positive for CMV DNA and negative for HSV 1 and 2, varicella zoster virus (VZV), and toxoplasmosis. Intravenous ganciclovir was prescribed but stopped after 3 weeks by an infectious disease specialist, who found no other signs of CMV disease.

**Figure 3:**
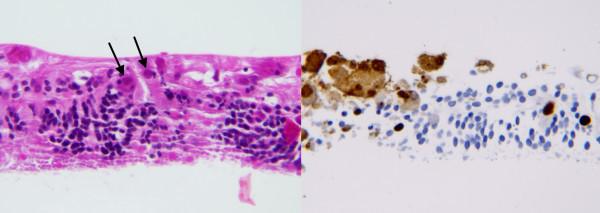
**Case 1, retinal biopsy. **Left frame: hematoxylin and eosin stain of retinal biopsy specimen showing cytomegalovirus inclusion bodies (arrows), ×100. Right frame: immunohistochemical stain with anti-cytomegalovirus antibody and avidin-biotin-peroxidase complex, 100X. Brown stain indicates retinal cells positive for cytomegalovirus antigens. Figures courtesy of Sander Dubovy, MD.

Three months later, the retinitis remained active. Vision was bare light perception, and the retinal circulation appeared more occluded. Diagnostic pars plana vitrectomy with retinal biopsy, membrane peel, and endolaser, and C_3_F_8_ intraocular gas tamponade was performed. PCR of vitreous fluid was again positive for CMV DNA and negative for HSV, VZV, toxoplasmosis, and Epstein-Barr virus. Retinal biopsy showed cytomegalic inclusion bodies and CMV by immunohistochemistry (Figure 3). At surgery, the nummular lesions appeared as multiple, intraretinal, white snowflake opacities with adhesion to the underlying RPE. No bleeding occurred during retinal incision. All retinal arteries were sclerotic, with a crystalline appearance, and the optic nerve head was completely atrophic. On final follow-up, the patient had a visual acuity of light perception in his right eye with neovascularization of the iris, ectropion uveae, and dense vitreous hemorrhage despite endolaser at surgery.

### Case 2

An 81-year-old white man experienced decreased vision and redness in his right eye that worsened over 3 weeks. Past medical history was significant for multiple sclerosis in 1950 with bilateral optic neuritis in remission, cerebrovascular accident in 2000, hyperlipidemia, and diabetes. Granulomatous anterior segment inflammation and a central retinal artery occlusion of the right eye were noted by an outside ophthalmologist. Serum CMV IgG antibody and varicella zoster antibody were positive. CMV IgM antibody, HSV I and II IgG antibodies, and syphilis antibody were negative. Carotid artery stenosis was 70% to 80% on the right and 50% on the left on carotid Doppler ultrasound. Varicella-related necrotizing retinitis was suspected, and famciclovir 500 mg three times daily was initiated.

**Figure 4:**
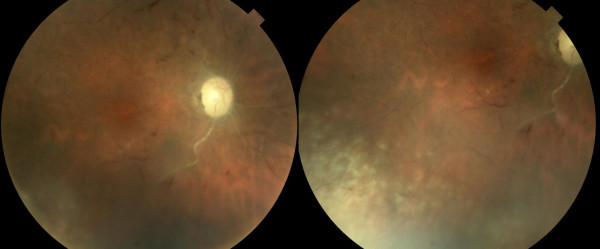
**Case 2, posterior fundus, right eye. **Left frame: 5 weeks after vision loss from central retinal artery occlusion, there is no recanalization of the artery. The venous blood columns are segmented with a few intraretinal hemorrhages. Retinitis is barely visible inferotemporal to the macula. Right frame: the inferotemporal periphery shows nummular lesions of a granular retinitis similar to Case 1. The vascular closure is generalized rather than specific to the area of retinitis.

On referral 2 months after onset, vision was counting fingers at 6 feet in the right eye and 20/20 in the left eye with a 2+ relative afferent pupillary defect in the right eye. There were large white keratic precipitates on the right corneal endothelium, 2+ anterior chamber cell, and 1+ flare. On dilated examination of the right eye, the optic nerve was pale, with sclerotic, attenuated arterioles, segmentation of the venous blood columns, and no evidence of recanalization (Figure 4). There was a patchy, granular retinitis in the inferotemporal quadrant. The diagnosis of necrotizing retinitis with central retinal artery occlusion was made. The left eye was free of inflammation.

Diagnostic AC paracentesis for PCR for CMV, varicella zoster virus, and herpes simplex virus was positive for CMV DNA only. HIV serology was negative. An intravitreal injection of foscarnet was given and oral valganciclovir was started. Three months later, there was florid neovascularization of the iris. The retinitis was inactive. Bevacizumab was injected intracamerally, and PRP was performed with regression of the NVI. Vision remained stable at hand motions. Valganciclovir was continued at 450 mg daily adjusted for renal function to complete 3 months of treatment.

### Case 3

A 66-year-old white man presented with 3 weeks of blurred vision and floaters in his left eye. Past medical history was significant for chronic open angle glaucoma and CLL status post chemotherapy, with last treatment 1.5 years prior. The patient denied pain, redness, or photophobia. Vision was 20/20- in the right eye and 20/30 in the left eye. The intraocular pressure was 20 mmHg in the right eye and 26 mmHg in the left eye. Slit lamp exam showed 2+ flare with rare cells and fine keratic precipitates in the left eye. Fundus examination on the left revealed 2+ vitreous cells. There was a small patch of chorioretinitis present in the superotemporal periphery. A presumptive diagnosis of toxoplasmosis chorioretinitis was made. Clarithromycin and atovaquone were started; laboratory studies included complete blood count with differential, complete metabolic profile, rheumatoid factor, ANCA, RPR, lyme antibodies, HLA-B27, and angiotensin converting enzyme. CBC was remarkable with 63,300 WBC with 92.4% lymphocytes. All other laboratory studies were within normal limits; HLA-B27 antigen was present. Subsequent toxoplasmosis IgG was positive, with a negative IgM.

**Figure 5:**
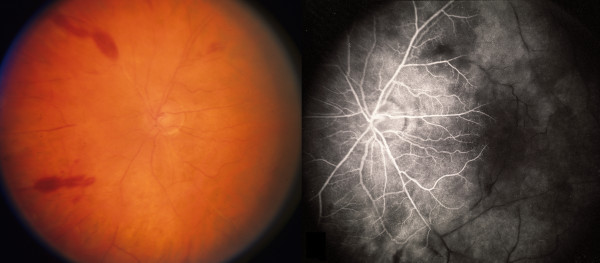
**Case 3, posterior fundus, left eye. **Left frame: the vasculature is perfused centrally but there are sheathed arterioles and intraretinal hemorrhages. Right frame: arteries and veins are truncated with extensive capillary nonperfusion in the macula.

**Figure 6:**
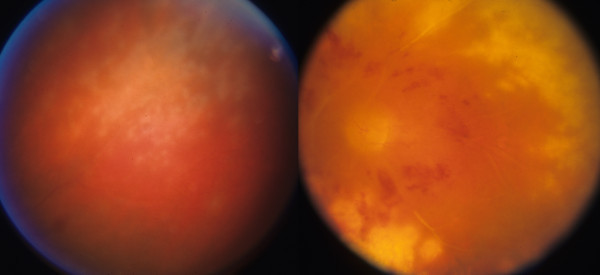
**Case 3, fundus, left eye. **Left frame: a single small focus of a granular necrotizing retinitis was present in the superotemporal retina at the time of central retinal artery occlusion. Right frame: typical CMV retinitis developed several weeks later after discontinuation of systemic anti-viral therapy with oral ganciclovir.

After 1 week, the retinitis appeared to resolve, but one month later, vision dropped to 20/200. Intraocular pressure was 32 mmHg with 1+ anterior chamber cell and flare and 2+ vitreous cells. Fundus examination showed extensive arteriolar closure with segmentation of the venous circulation confirmed by fluorescein angiography (Figure 5). The focus of chorioretinitis superotemporally had enlarged slightly (Figure 6, left). Diagnostic vitrectomy was performed 2 days later; vision prior to surgery had declined to 6/200 ‘E’. The vitrectomy was combined with trabeculectomy with mitomycin C. Vitreous fluid was sent for PCR analysis for CMV, HSV 1 and 2, and VZV DNA, as well as toxoplasma IgG, flow cytometry and cytology, and viral culture. All of these tests were negative.

Empiric valacyclovir was started. The retinal vessels appeared attenuated with macular ischemic whitening consistent with central retinal artery occlusion. Two weeks later, vision declined to 3/200 ‘E’. Anterior chamber paracentesis was performed, and CMV DNA was detected by PCR. Intravenous ganciclovir was started 300 mg every 12 h for 14 days followed by oral ganciclovir 1 gram three times daily. Within 2 months, the retinitis appeared healed; however, neovascular glaucoma developed requiring Baerveldt glaucoma drainage implant. The intraocular inflammation and retinitis reactivated, with focal nummular white lesions encroaching on the superior arcade. No further therapeutic intervention was recommended due to poor visual prognosis, and the retinitis eventually progressed to involve most of the periphery (Figure 6, right).

## Discussion

The pathogenesis of CMVR likely involves hematogenous dissemination with initial infection of the vascular endothelium and subsequent spread into adjacent tissues [5]. In HIV infection, breakdown of the blood-retinal barrier by HIV retinopathy [6] may facilitate viral spread into the retinal tissue. Pro-inflammatory cytokines may contribute to the vasculopathic effects of the virus [7] with expression of endothelial adhesion molecules, leading to increased platelet and leukocyte adhesion, induction of anti-phospholipid antibodies, and enhanced factor VIII synthesis and secretion [7].

CMVR and HIV-related branch retinal artery occlusions have been rarely reported [8,9]. There have also been a few case reports of retinal vascular occlusion in HIV/AIDS patients without CMVR [10-15]. In the five cases of ARN-like CMVR in immunocompetent patients reported in the literature [2,3,16,17], one had branch retinal artery occlusion [14]. Designation of patients as immunocompetent or immunocompromised is imprecise even when special testing of the immune system is performed. The elderly patients we deemed immunocompetent were not receiving immunosuppression for organ transplantation or chemotherapy for cancer, which are the usual causes for CMVR in non-HIV patients. They were elderly with associated co-morbidities and, therefore, in a gray zone of immunocompetency. They represent a large population at risk for CMVR with few apparent risk factors other than advanced age.

Well-established risk factors for vascular occlusion were present in our patients including hypertension, increased white blood cell counts, uncontrolled glaucoma, giant cell arteritis, cardiovascular disease, and carotid stenosis [18]. CMV infection has been associated with atherosclerosis and with impaired circulation [19,20]. Vascular occlusion contributed to poor visual acuity outcomes independent of macular involvement or retinal detachment, which are the usual means by which patients with CMVR lose vision but were not present in these patients. A weaker than normal immune system in these elderly patients that allowed for CMVR combined with a sufficient immune response to damage and close vessels moderately impaired by other vasculopathies may have produced this unique presentation. In cases 1 and 4, the early use of corticosteroids without antiviral cover before the diagnosis of CMVR was made may have allowed sufficient worsening to permit this unusual constellation of findings.

Extensive vascular occlusion occurred when the retinal surface area involved with retinitis was still quite small. Although risk factors for vascular closure were present, the artery occlusions were not typical for embolic disease, for example, typical cherry-red spots and recanalization did not occur. Despite extensive occlusions as in cases 1 and 3, at least some portion of the central retinal circulation remained patent until late in the course. The process therefore was more likely progressive nonperfusion that finally affected the central vasculature rather than an acute event such as a central retinal artery occlusion. The incremental closure of the total retinal circulation may have led to other atypical features such as the high frequency of secondary neovascularization, which is unusual in central retinal artery occlusion and also unusual in eyes of patients with CMVR involving the entire retina.

## Conclusion

Retinal artery occlusion can occur in conjunction with CMVR in elderly, immunocompetent patients. Recognition of CMV as a rare cause of necrotizing retinitis and institution of early antiviral treatment may reduce the risk of this devastating complication. Since generalized vascular occlusion is an uncommon manifestation of CMVR, older age, concurrent illnesses, and relatively intact immune defenses with intravascular inflammation may have contributed to this devastating outcome.

## Methods

Patient lists were searched to identify non-HIV-infected patients with CMVR from 2000–2007. Medical records were retrospectively reviewed for age and sex; medical status including iatrogenic immunosuppression; ocular characteristics including extent of retinitis, vascular occlusions, neovascularization, and hemorrhage; clinical estimate of grade of inflammation; clinical course including timing of vascular occlusion relative to onset of symptoms; results of diagnostic studies; medical treatment and response to medication; surgical interventions; and visual outcomes. Narrative accounts of three cases considered to be immunocompetent who developed retinal arteriolar occlusion were created. Control patients were selected from a group of HIV-infected patients with CMVR confirmed by polymerase chain reaction (PCR) testing and previously reported from this institution [21].

Paired McNemar statistics were used to compare the presence and absence of bilateral disease, profound vision loss, arteriolar occlusion, neovascularization or vitreous hemorrhage, and retinal detachment between the non-HIV-infected and HIV-infected patients. Small sample size hampered comparisons between the elderly, immunocompromised and elderly, immunocompetent groups, and the elderly, immunocompetent and HIV-infected groups. Expedited approval for retrospective review of medical records was granted by the Human Subjects Review Board by the University of Miami Miller School of Medicine with waiver of HIPAA authorization.

## Competing interest

The authors declare that they have no competing interests.

## Authors’ contributions

JLD conceived of the study, provided patients, performed the statistical analysis, and wrote the final manuscript. PH and KH provided patients and drafted the manuscript. All authors read and approved the final manuscript.

## Authors’ information

The authors presented this study at the Joint Meeting Retina Society, Macula Society, American Society of Retina Specialists, New York, NY on October 2, 2009. KH and PH participated in the study while in residency training at Bascom Palmer Eye Institute and are now practicing ophthalmologists.

## References

[B1] Staras SA Dollard SC Radford KW Flanders WD Pass RF Cannon MJ Seroprevalence of cytomegalovirus infection in the United States, 1988–1994 Clin Infect Dis 2006 43 1143 1151 10.1086/508173 17029132

[B2] Long HM Dick A Presumed CMV associated necrotizing retinopathy in a non-HIV immunocompromised host Clin Experiment Ophthalmol 2005 33 330 332 10.1111/j.1442-9071.2005.00996.x 15932541

[B3] Voros GM Pandit R Snow M Griffiths PG Unilateral recurrent acute retinal necrosis syndrome caused by cytomegalovirus in an immune-competent adult Eur J Ophthalmol 2006 16 484 486 16761257 10.1177/112067210601600323

[B4] Stewart MW Bolling JP Mendez JC Cytomegalovirus retinitis in an immunocompetent patient Arch Ophthalmol 2005 123 572 574 10.1001/archopht.123.4.572 15824240

[B5] Rao NA Zhang J Ishimoto S Role of retinal vascular endothelial cells in development of CMVR Trans Am Ophthalmol Soc 1998 96 111 123 10360285 PMC1298391

[B6] Glasgow BJ Evidence for breaches of the retinal vasculature in acquired immune deficiency syndrome angiopathy A fluorescent microsphere study Ophthalmology 1997 104 753 760 10.1016/s0161-6420(97)30237-1 9160019

[B7] Abgueguen P Delbos V Chennebault JM Payan C Pichard E Vascular thrombosis and acute cytomegalovirus infection in immunocompetent patients: report of 2 cases and literature review Clin Infect Dis 2003 36 134 139 10.1086/374664 12766855

[B8] Roarty JD Fisher EJ Nussbaum JJ Long-term visual morbidity of cytomegalovirus retinitis in patients with acquired immune deficiency syndrome Ophthalmology 1993 100 1685 1688 8233395 10.1016/s0161-6420(93)31417-x

[B9] Conway MD Tong P Olk RJ Branch retinal artery occlusion (BRAO) combined with branch retinal vein occlusion (BRVO) and optic disc neovascularization associated with HIV and CMVR Int Ophthalmol 1995–1996 19 249 252 10.1007/BF00132694 8737706

[B10] Friedman SM Margo CE Bilateral central retinal vein occlusions in a patient with acquired immunodeficiency syndrome. Clinicopathologic correlation Arch Ophthalmol 1995 113 1184 1188 10.1001/archopht.1995.01100090110031 7661754

[B11] Teich SA Sonnabend J Central retinal vein occlusion in a patient with AIDS. Case report Arch Ophthalmol 1988 106 1508 1509 10.1001/archopht.1988.01060140676016 3190534

[B12] Ismail Y Nemechek PM Arsura EL A rare cause of visual loss in AIDS patients: central retinal vein occlusion Br J Ophthalmol 1993 77 600 601 10.1136/bjo.77.9.600 8218062 PMC513961

[B13] Wen F Chen X Liao R Bilateral central retinal vein occlusions in a Chinese patient with HIV-infection Int Ophthalmol 2001 24 173 175 10.1023/A:1021137423674 12498514

[B14] Dunn JP Yamashita A Kempen JH Jabs DA Retinal vascular occlusion in patients infected with human immunodeficiency virus Retina 2005 25 759 766 10.1097/00006982-200509000-00012 16141865

[B15] Park KL Marx JL Lopez PF Rao NA Noninfectious branch retinal vein occlusion in HIV-positive patients Retina 1997 17 162 164 9143047

[B16] Digre KB Blodi CF Bale JF Cytomegalovirus infection in a healthy adult associated with recurrent branch retinal artery occlusion Retina 1987 7 230 232 10.1097/00006982-198707040-00006 2829330

[B17] Saidel MA Berreen J Margolis TP Cytomegalovirus retinitis after intravitreous triamcinolone in an immunocompetent patient Am J Ophthalmol 2005 140 1141 1143 10.1016/j.ajo.2005.06.058 16376669

[B18] Recchia FM Brown GC Systemic disorders associated with retinal vascular occlusion Curr Opin Ophthalmol 2000 11 462 467 10.1097/00055735-200012000-00013 11141642

[B19] Nerheim PL Meier JL Vasef MA Li WG Hu L Rice JB Gavrila D Richenbacher WE Weintraub NL Enhanced cytomegalovirus infection in atherosclerotic human blood vessels Am J Pathol 2004 164 589 600 10.1016/S0002-9440(10)63148-3 14742264 PMC1602282

[B20] Grahame-Clarke C Chan NN Andrew D Ridgway GL Betteridge DJ Emery V Colhoun HM Vallance P Human cytomegalovirus seropositivity is associated with impaired vascular function Circulation 2003 108 678 683 10.1161/01.CIR.0000084505.54603.C7 12900349

[B21] Harper TW Miller D Schiffman JC Davis JL Polymerase chain reaction analysis of aqueous and vitreous specimens in the diagnosis of posterior segment infectious uveitis Am J Ophthalmol 2009 147 140 147 10.1016/j.ajo.2008.07.043 18834576 PMC4142712

